# Small-Molecule Ferroptotic Agents with Potential to Selectively Target Cancer Stem Cells

**DOI:** 10.1038/s41598-019-42251-5

**Published:** 2019-04-11

**Authors:** William R. Taylor, Sara R. Fedorka, Ibtissam Gad, Ronit Shah, Hanan D. Alqahtani, Radhika Koranne, Nishanth Kuganesan, Samkeliso Dlamini, Tim Rogers, Ayad Al-Hamashi, Veronika Kholodovych, Yusuf Barudi, Damian Junk, Maisha S. Rashid, Mark W. Jackson, L. M. Viranga Tillekeratne

**Affiliations:** 1 Department of Medicinal and Biological Chemistry, College of Pharmacy and Pharmaceutical Sciences, The University of Toledo, 2801 W. Bancroft Street, Toledo, Ohio 43606 United States; 2 Department of Biological Sciences, College of Natural Sciences and Mathematics, The University of Toledo, 2801 W. Bancroft Street, Toledo, Ohio 43606 United States; 3 Department of Pathology, Case Western Reserve University School of Medicine, Case Comprehensive Cancer Center, 2103 Cornell Road, WRB 3-134, Cleveland, Ohio 44106 United States

## Abstract

Effective management of advanced cancer requires systemic treatment including small molecules that target unique features of aggressive tumor cells. At the same time, tumors are heterogeneous and current evidence suggests that a subpopulation of tumor cells, called tumor initiating or cancer stem cells, are responsible for metastatic dissemination, tumor relapse and possibly drug resistance. Classical apoptotic drugs are less effective against this critical subpopulation. In the course of generating a library of open-chain epothilones, we discovered a new class of small molecule anticancer agents that has no effect on tubulin but instead kills selected cancer cell lines by harnessing reactive oxygen species to induce ferroptosis. Interestingly, we find that drug sensitivity is highest in tumor cells with a mesenchymal phenotype. Furthermore, these compounds showed enhanced toxicity towards mesenchymal breast cancer populations with cancer stem cell properties *in vitro*. In summary, we have identified a new class of small molecule ferroptotic agents that warrant further investigation.

## Introduction

Despite major advances in cancer prevention and early detection, many tumors are only detected after they have spread to distant organs. In fact, analysis of some human tumors suggests that dissemination may occur very early while the primary tumor is small and difficult to detect^1^. Systemic treatments for disseminated cancer, including new small chemotherapeutic molecules, are needed to combat this disease. An ideal cancer therapy will destroy tumor deposits leaving normal tissues unharmed. Therefore, recent approaches to drug design and discovery include targeted compounds that take advantage of genetic changes and targets unique to the tumor cell. One of these approaches targets cells harboring mutant Ras oncoproteins which are activated in a wide variety of human cancers.

Erastin is the prototype of recently discovered Ras-selective lethal (RSL) compounds that more efficiently kill cells harboring activated Ras alleles^2–5^. These compounds do not directly target the mutant Ras protein, but take advantage of aspects of tumor cell metabolism associated with the Ras-transformed state for selective killing. These drugs induce the accumulation of reactive oxygen species (ROS) to which Ras-transformed cells are highly sensitive^2–5^. Cell death induced by erastin occurs in a unique manner, independent of caspase activity, but highly dependent on iron. This mode of cell death, called ferroptosis, is rapid and likely occurs by catastrophic peroxidation of membrane lipids^3,6–10^.

**Figure 1:**
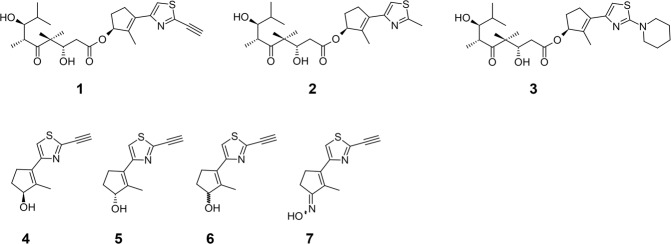
Small molecule anticancer agents.

We recently reported that in the course of developing open chain epothilone analogues, we discovered the molecule **1** (Fig. 1) that killed selected cancer cells by a non-apoptotic mechanism of action^11^. Its analogues, compounds **2** and **3** without a terminal alkyne group were not active. The observed cytotoxic activity of compound **1** was in fact, found to be due to its hydrolysis product **4** (Fig. 1) which had a similar activity profile as the parent compound **1**. Further investigations revealed that compound **4**, its epimer **5**, the racemate **6**, and the oxime analogue **7** selectively killed cancer cells by a nonapoptotic mechanism of action mediated by ROS in an iron-dependent manner, consistent with ferroptosis^11^. Here we further characterize the mechanism of cell death, and identify a potential mechanism of action. Of note, by analyzing a number of normal and cancer cell lines, we found that compound sensitivity correlates with a mesenchymal state. Re-expression of E-cadherin in the sensitive lung cancer cell line NCI-H522 reduced compound sensitivity while knocking E-cadherin out of HCT116 cells sensitized them. In addition, toxicity is enhanced in a mesenchymal breast cancer subpopulation with cancer stem cell (CSC) properties. Therefore, the compounds we describe may represent a new approach to target CSCs for more efficient tumor killing.

## Results

### Ferroptotic cell death in cells exposed to compound 4

**Figure 2:**
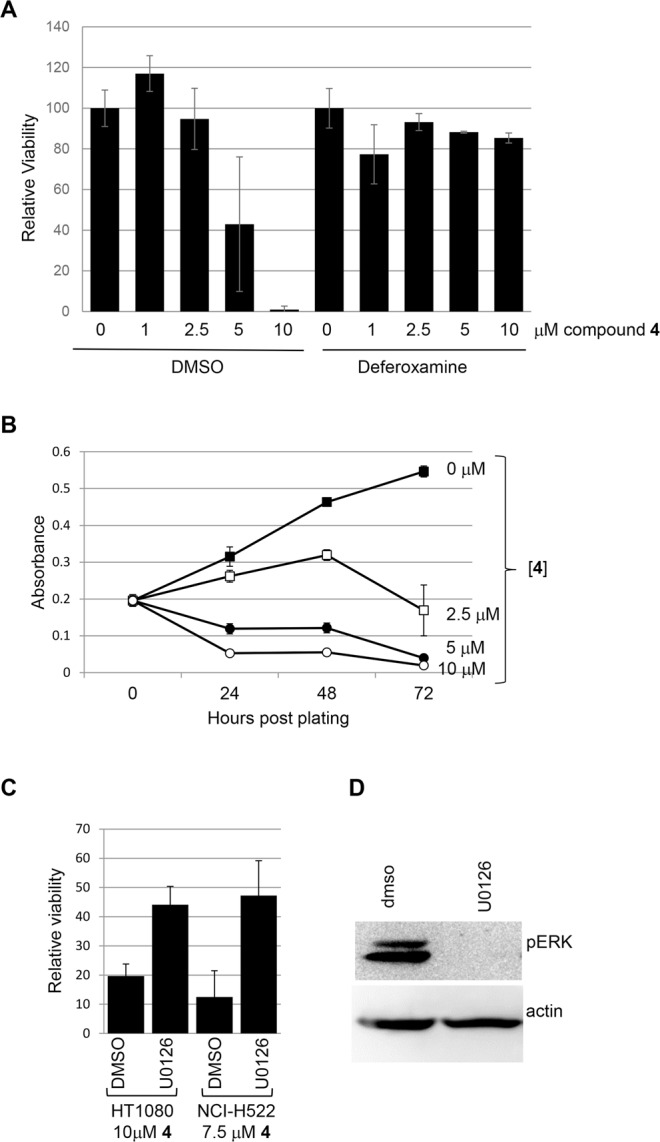
Compound **4** toxicity requires iron and is inhibited by U0126. (**A**) NCI-H522 cells were exposed to the indicated concentrations of **4** in the presence or absence of deferoxamine. Bars = standard deviation. (**B**) Dose and time-dependency of compound **4** toxicity was tested on NCI-H522. (**C**) Inhibiting MEK1/2 reduced killing by **4** in HT1080 cells and NCI-H522. (**D**) Erk phosphorylation in NCI-H522 cells. Western blotting to detect phosphorylated Erk in the presence or absence of U0126 is shown. Actin is included as a loading control. To measure viability, cells were exposed to drugs for the times indicated and then stained with methylene blue.

Initial studies with compound **4** suggested that cytotoxicity towards a number of cancer cells was due to induction of ferrotposis. For example, killing of NCI-H522 cells by compound **4** was blocked by iron chelators ciclopirox olamine^11^, hydroxyurea^11^ and deferoxamine (Fig. 2A). In sensitive cell lines, cell death was rapid with most cells dead by one day after treatment (Fig. 2B). LD50s were in the low micromolar range (Fig. 2B and ref.^11^). Sensitivity was enhanced by adding ferric citrate, suggesting that iron plays a role in cell death mechanism^11^. Further, free radical scavengers, trolox and butylated hydroxyanisole blocked the death of NCI-H522 cells induced by **4**, suggesting that compound **4** relies on ROS for cytotoxic activity^11^. Cell death can be attributed to damage to proteins, lipids, and nucleic acids caused by elevation of ROS beyond the antioxidant capacity of the cell^12^. Cellular sources of ROS include incomplete reduction of O_2_ during electron transport to form superoxide and direct generation of superoxide by the membrane bound NADPH oxidases^12^. Intracellular ROS was elevated in NCI-H522 cells exposed to compound **4**. Also, ROS elevation by **4** was dependent on iron and NADPH oxidase and was blocked by trolox^11^.

### Mechanism of action of compound **4**

Ferroptosis was originally described as a response to the RSL compound erastin^3^. Erastin preferentially kills Ras-transformed cells, and blocking MEK1 and 2 with U0126 reduces killing^3,13^. Compound **4** killed HT1080 cells harboring an activated N-Ras (Fig. 2C). Compound **4** also killed NCI-H522 cells that have elevated ERK phosphorylation despite containing wild-type Ras alleles (Fig. 2D)^14,15^. Killing of both cell lines was reduced by co-treatment with U0126 (Fig. 2C). However, U0126 was recently shown to act as an antioxidant, therefore, its effects on **4** toxicity may not be strictly due to inhibition of Ras signaling^16,17^. Future experiments will directly test whether Ras signaling modulates killing by compound **4**.

**Figure 3:**
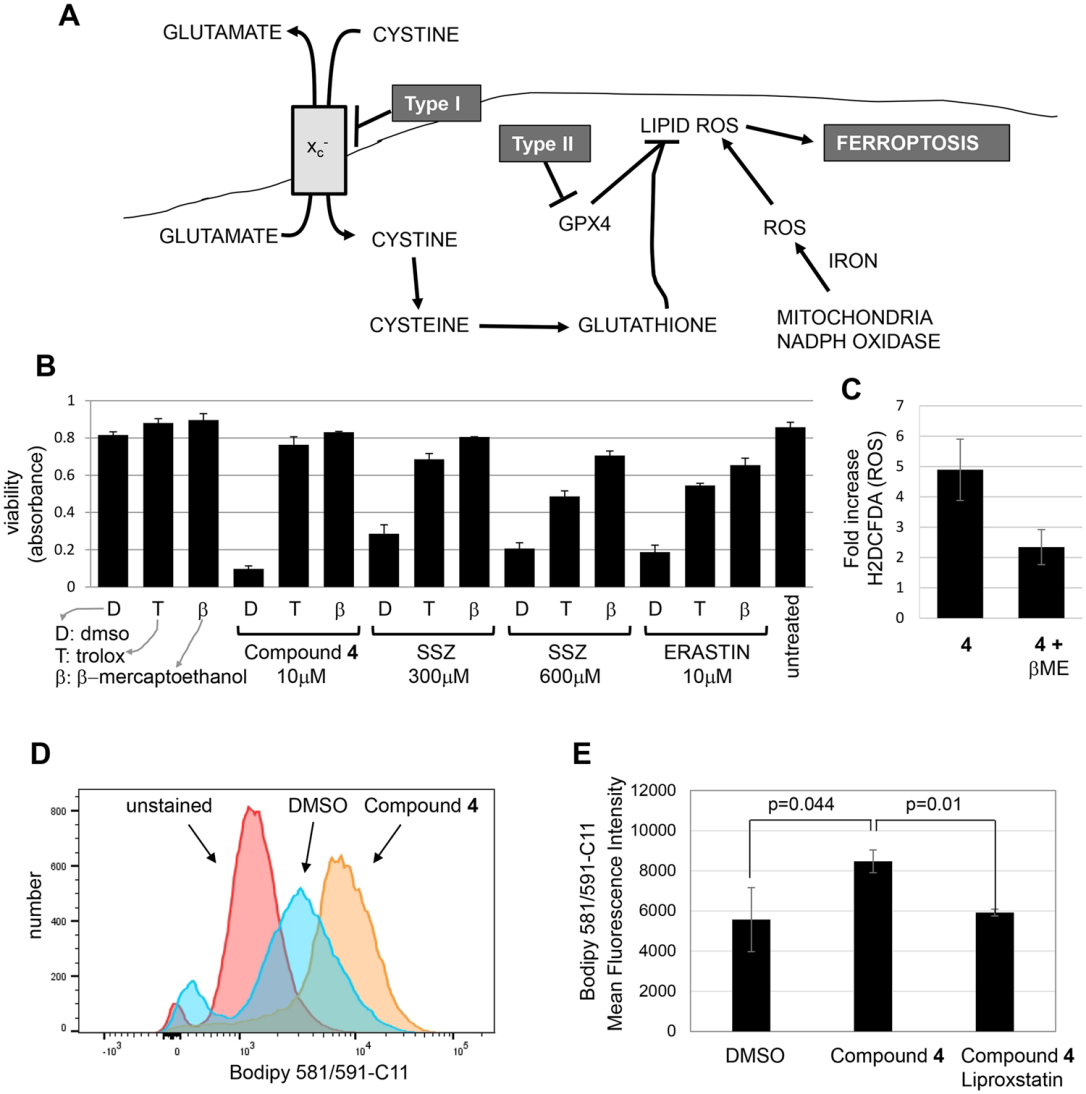
Compound **4** induces ferroptosis. (**A**) Overview of ferroptosis (as described in^5^). (**B**) Similar effects of compound **4**, erastin, and sulfasalazine (SSZ) on NCI-H522 cells. Cells were exposed to the compounds indicated and viable cells quantified 2 days later using methylene blue. (**C**) Elevation of ROS in NCI-H522 cells exposed to 10 μM compound **4** is blocked by β-mercaptoethanol (βME). Cells were exposed to the indicated compounds for 4 hours and stained with H2DCFDA. Stain was extracted and quantified using a fluorescence plate reader. (**D**) Measurement of lipid oxidation. NCI-H522 cells were exposed to DMSO or compound **4** for 10 hours. Bodipy 581/591-C11, a membrane bound ROS sensor was added at the time compounds were added. Fluorescence was detected by FACS and mean fluorescent intensities of triplicate samples compiled in part (**E**). As expected, the lipid ROS scavenger liproxstatin blocked the oxidation of the dye in response to **4**. Bars throughout represent averages and associated standard deviations.

Ferroptosis appears to be a response to elevated lipid ROS resulting in loss of membrane integrity (Fig. 3A)^5,18^. Lipid ROS are detoxified in a reaction catalyzed by GPX4 using glutathione as a reducing agent. Therefore, ferroptosis can be triggered by depleting glutathione or inhibiting GPX4 and drugs that induce ferroptosis can be classified according to their specific targets^3,5^. Type I ferroptosis drugs (like erastin and sulfasalazine) block the x_c_^−^ amino acid transporter which imports cystine needed for glutathione synthesis. Type II compounds (like RSL3) directly inhibit GPX4 (Fig. 3A)^3,5^. ROS in the form of superoxide may form via the activity of NADPH oxidases, or via the incomplete reduction of oxygen during electron transport in the mitochondria (Fig. 3A). An early study indicated that ferroptosis could occur in cells lacking mitochondria, suggesting that superoxide produced by NADPH oxidases was sufficient to induce the process^3^. In another study however, mutation of GPX4 was found to sensitize to inhibitors of mitochondrial complex I, suggesting that mitochondria may contribute to ferroptosis^19^.

Ferroptosis induced by Type I compounds can be inhibited by β-mercaptoethanol (βME). One interpretation of the protective effect of βME suggests that since it forms mixed disulfides with cystine, this will release reduced cysteine that enters the cell via alternative amino acid transporters, thereby bypassing x_c_^− ^^7^_._ βME abrogated killing of NCI-H522 cells and reduced ROS in response to **4** (Fig. 3B,C). Both erastin and sulfasalazine killed NCI-H522 cells in a manner that was blocked by trolox and βME (Fig. 3B)^20^. These data suggest that NCI-H522 cells require x_c_^−^ for survival and provide one piece of evidence that compound **4** may kill cells by the type I mechanism. It is also important to note that βME may protect cells by providing reducing equivalents downstream of the cystine transporter, perhaps by acting as a Gpx4 substrate. Therefore, the effects of βME only provide indirect evidence for the mechanism of action of compound **4**. Oxidation of membrane lipids during ferroptosis can be detected with the membrane-bound ROS sensor dye Bodipy 581/591-C11. Treatment with compound **4** increased Bodipy 581/591 fluorescence providing additional evidence of ferroptosis (Fig. 3D,E). Fluorescence was reduced by co-treatment with the lipid ROS scavenger liproxstatin indicating that the assay is specific (Fig. 3E).

**Figure 4:**
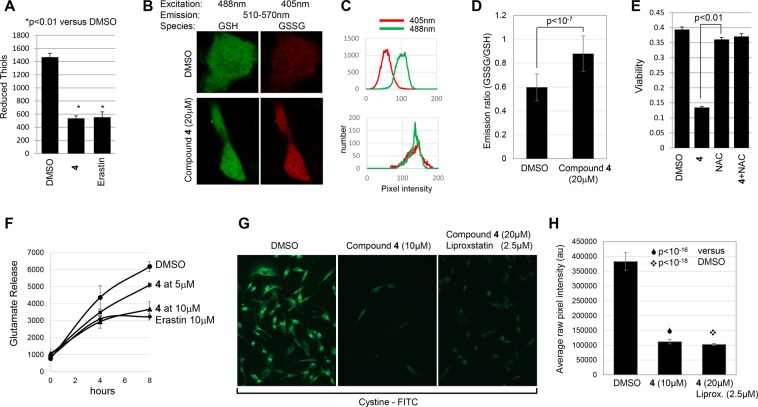
Compound **4** is a Type I inhibitor. (**A**) **4** depletes reduced thiols. NCI-H522 cells were exposed to either **4** or erastin. Thiols were measured 6 hours later using monochlorobimane. (**B**) Oxidation of a glutathione biosensor in cells exposed to compound **4**. HT1080 cells were transiently transfected with Grx1-roGFP2, exposed to 20 μM compound **4** for 8 hours and subject to live-cell confocal imaging. Examples of scanned cells are shown in (**B**), with pixel intensities of representative cells shown in (**C**). Excitation and emission settings are shown in the figure. In (**D**), the ratio of pixel intensities excited at 405 nm versus 488 nm were compiled for at least 45 cells. (**E**) **4**-induced cell death is blocked by N-acetylcysteine (NAC). NCI-H522 cells were exposed to the indicated compounds for three days. Viability was determined by methylene blue staining. (**F**) **4** inhibits glutamate release. HT1080 cells exposed to the compounds indicated were incubated in medium lacking glutamate for up to 8 hours. Conditioned media were assayed for secreted glutamate. At 8 hrs, 5 μM and 10 μM compound **4** were significantly lower than DMSO (p value of 0.01 and 0.002 respectively). (**G**) **4** inhibits Cystine-FITC uptake. MDA-MB-231 cells were exposed to **4** with or without liproxstatin (2.5 μM) for 16 hours. Cystine-FITC was added at 5 μM and images collected 1 hour later. Pixel intensities from digital images of at least 70 cells are compiled in (**H**).

A characteristic feature of Type I but not Type II inhibitors is that they deplete glutathione^5^. One method to measure reduced glutathione is to use monochlorobimane which forms a fluorescent adduct with reduced glutathione. NCI-H522 showed a similar depletion of monochlorobimane staining when exposed to either erastin or **4** (Fig. 4A). Monochlorobimane can react less efficiently with non-glutathione thiols and therefore does not provide an unequivocal measure of glutathione content^21^. Next, we used the Grx1-roGFP2 biosensor to independently measure reduced glutathione. In this system, Grx1 in close proximity to roGFP2 uses glutathione to reduce cysteines engineered into the GFP moiety. Reduction shifts the excitation spectrum allowing reduced and oxidized forms of roGFP2 to be distinguished by confocal imaging^22^. Using this system we observed a modest but significant increase in the reduced form of roGFP2 upon treatment with compound **4**, suggesting that this compound depletes reduced glutathione (Fig. 4B–D). Next, we reasoned that if **4** were a Type I inhibitor, providing cysteine from an external source would also block killing. Consistent with this idea, N-acetyl-cysteine, which is converted to reduced cysteine intracellularly, was capable of blocking **4**-induced cell death (Fig. 3E).

These various lines of evidence suggest that compound **4** is a type I ferroptosis inducer. To test this idea directly, we analyzed activity of the x_c_^−^ transporter. We observed a reduction in glutamate secretion from cells exposed to compound **4** (Fig. 4F). Since x_c_^−^ is a cystine/glutamate antiporter, these data suggest that **4** inhibits x_c_^−^. We also observed that compound **4** reduced the uptake of cystine-FITC (Fig. 4G,H), a fluorescent dye previously shown to enter cells via x_c_^− ^^23^. Together, these data strongly suggest that compound **4** is a Type I inhibitor that induces ferroptosis by blocking x_c_^−^ function.

### Comparison of compound 4 to erastin

**Figure 5:**
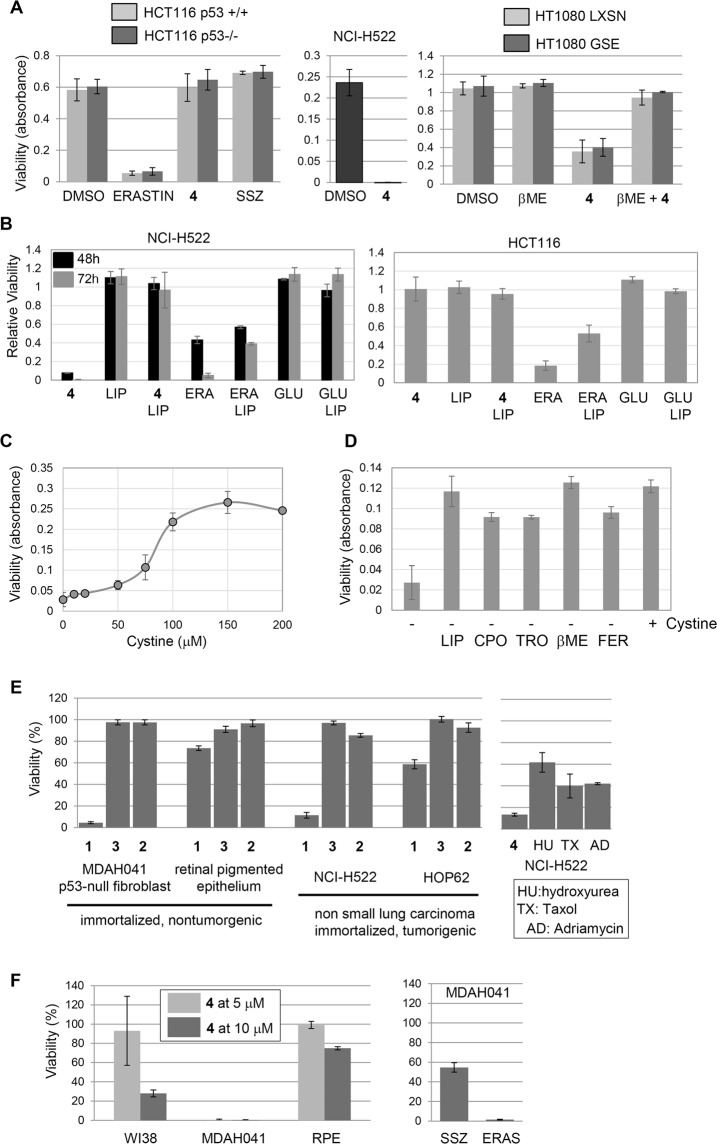
Drug sensitivity in various cell types. Cells were treated as indicated and viability determined by methylene blue staining. Bars represent averages and associated standard deviations. (**A**) Drug sensitivity in cancer cell lines differing in p53 status. Parental HCT116 with wild-type p53 were compared to p53-null HCT116 cells. Cells were exposed to 10 μM erastin, 10 μM compound **4**, or 300 μM sulfasalazine for 3 days before viability was determined. **4** was tested in parallel on NCI-H522 to confirm activity. **4** toxicity was also assessed in parental HT1080 cells with wild-type p53, and cells overexpressing a dominant negative p53 fragment, GSE56. Cells were exposed to 10 μM compound **4** in the presence or absence of βME and viability determined. (**B**) Effect of Liproxstatin. NCI-H522 and HCT116 cells were exposed to 10 μM of either compound **4** or Erastin in the presence or absence of 2.5 μM liproxstatin. Viability was determined 3 days later. Excess glutamate (5 mM) had no effect on cell viability. (**C**) NCI-H522 cells depend on external cystine. Cells were grown in medium lacking cystine supplemented with dialyzed fetal bovine serum for 3 days. Additional compounds were added and viability determined. (**D**) Dose response of external cystine. NCI-H522 cells were grown for 3 days in the indicated concentrations of cystine and viability determined. (**E**) Effects of **1** and analogues on cell viability. The cell types indicated were exposed to the indicated analogues for 2 days and viability determined. MDAH041 are a spontaneously immortalized p53-null human fibroblast cell line^51,52^. NCI-H522 were exposed to classical chemotherapy drugs for 2 days (HU: 2 mM, TX: 10 μM, AD: 0.2 μg/ml). (**F**) Effects of various compounds on WI38, MDAH041 and retinal pigmented epithelial (RPE) cells. Cells were exposed **4**, sulfasalazine (300 μM) or erastin (10 μM) 3 days and viability determined. (SSZ: sulfasalazine; βME: β-mercaptoethanol; LIP: liproxstatin; ERA: erastin; GLU: glutamate; CPO: ciclopirox olamine; TRO: trolox; FER: ferrostatin).

Compound **4** did not completely mimic the effects of erastin. For example, HCT116 colon cancer cells, which are sensitive to erastin, were relatively insensitive to **4** (Fig. 5A). We previously estimated that compound **4** kills HCT116 with an LC_50_ of ~76 μM^11^. Therefore, these compounds may have non-overlapping intracellular targets. Relevant to this idea is the fact that in addition to inhibiting x_c_^−^, erastin also binds to and inhibits the mitochondrial VDAC channel which may contribute to its mechanism of death^24^. As an additional comparison between compound **4** and erastin, we tested the effect of the ferroptosis inhibitor liproxstatin^25^. Liproxstatin completely blocked killing of NCI-H522 cells by compound **4** (Fig. 5B). However, 50% of HCT116 cells were still killed when exposed to erastin plus liproxstatin (Fig. 5B). It is possible that this residual killing is due to VDAC inhibition.

To further investigate the connection between ferroptosis in NCI-H522 cells and cystine availability, we deprived them of the amino acid. Cells died without cystine, but could be rescued by chelating iron (cyclopirox olamine), scavenging ROS (Trolox, ferrostatin), scavenging lipid ROS (liproxstatin) or providing reduced thiols (βME) (Fig. 5C,D). Maximal rescue occurred at 100 μM external cysteine (Fig. 5D). These results indicate that NCI-H522 can be induced to undergo ferroptosis simply by removing cystine. Next, we attempted to induce ferroptosis in NCI-H522 by elevating external glutamate to interfere with the x_c_^−^ antiporter activity. Adding 5 mM glutamate for three days had no effect on viability (Fig. 5B).

The highly sensitive NCI-H522 cell line harbors mutant p53^26^. To test the role of p53 status in **4** sensitivity, we analyzed two isogenic tumor lines differing in p53 status. Loss of p53 by gene targeting did not sensitize HCT116 colon cancer cells to compound **4** (Fig. 5A)^27^. Also, interfering with p53 using a dominant-negative fragment (GSE56) did not further sensitize the HT1080 fibrosarcoma cell line (Fig. 5A)^28^. Therefore, p53 status in these tumor lines does not modulate sensitivity to **4**. In short-term (2 day) assays, NCI-H522 cells were more efficiently killed by compound **4** compared to the classical drugs hydroxyurea, taxol, and adriamycin (Fig. 5E). At later time points, all four compounds killed NCI-H522 cells. The difference at early time points illustrates the speed of killing by compound **4**. Further, as we have described in more detail elsewhere, specific molecular determinants were essential for activity. In particular, replacement of the terminal alkyne moiety on the thiazole ring with other groups eliminated activity (Figs 1, 5E and ref.^11^).

### Sensitivity of mesenchymal cells to compound 4

Comparison of a number of normal and cancer cell lines and strains showed that **4** and structurally related compounds were selective with cells varying ~100 fold in sensitivity^11^. In this initial study we surveyed overall sensitivity in 19 different human cell lines representing normal tissues, as well as cancers of the lung, cervix, muscle, colon, breast and prostate. One of the factors influencing sensitivity appeared to be the mesenchymal state. For example, HT1080 fibrosarcoma, Rh30 and Rh41 rhabdomyosarcoma, as well as immortalized p53-null fibroblasts MDAH041 were sensitive, whereas epithelial cells (MCF7, HCT116, retinal pigmented epithelium:RPE) were not easily killed by these compounds (Fig. 5E and ref.^11^). Similar to **1**, compound **4** killed the mesenchymal WI38 and MDAH041 cells more efficiently than the RPE cells (Fig. 5F). WI38, a non-immortalized diploid human cell strain with normal p53 function, was more resistant than the immortalized p53-null MDAH041 cell line. Given that p53 status did not affect two other cancer cell lines, it is possible that the immortalized state of MDAH041 renders them more sensitive than WI38 cells. Interestingly, MDAH041 cells were also sensitive to the ferroptotic agents sulfasalazine and erastin (Fig. 5C).

**Figure 6:**
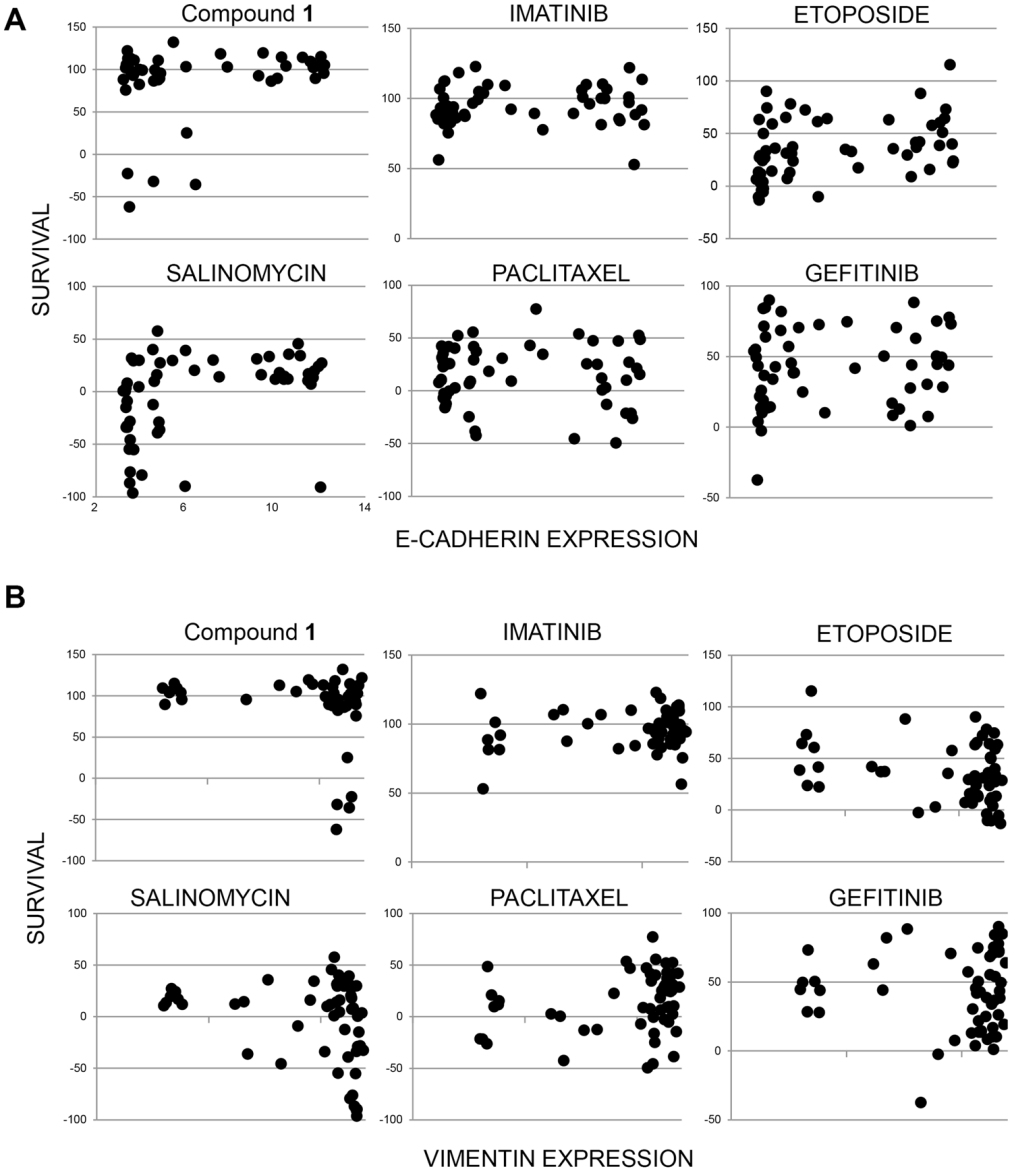
Mesenchymal markers correlate with compound sensitivity. (**A**) Correlation of low E-cadherin with drug sensitivity. Survival data with **1** was obtained from our own 60 cell line NCI screen. Other compounds were queried using CellMiner to obtain NCI-60 drug sensitivity data sets^53,54^. E-Cadherin (cdh1) expression levels were obtained from NCBI (Geo Dataset GDS4296)^55^. (**B**) Drug sensitivity correlates with high vimentin expression. Expression and survival data was collected as described in “A”.

Using data from our 60 cell line NCI screen, we tested whether sensitivity correlated with expression of E-cadherin, an epithelial marker. All of the sensitive cell lines had low E-cadherin, but not all of the low E-cadherin cell lines were sensitive (Fig. 6A). Therefore, low expression of E-cadherin appears to be necessary but not sufficient for **1** sensitivity in tumor cell lines. Sensitivity to classical chemotherapy drugs paclitaxel and etoposide, as well as imatinib (BCR-Abl inhibitor), and gefitinib (EGFR inhibitor) did not correlate with E-cadherin expression (Fig. 6A). Salinomycin, selected in a screen for drugs that kill cells that do not express E-cadherin, showed a strong correlation in our comparison. With one exception, only low E-cadherin expressing cell lines were killed by salinomycin (Fig. 6A). When comparing drug toxicity to expression of the mesenchymal marker vimentin, the opposite correlations were observed. Compound **1** only killed cells with high vimentin, and most salinomycin-sensitive cell lines had high vimentin. Sensitivity to paclitaxel, etoposide, imatinib and gefitinib did not correlate with vimentin expression (Fig. 6B).

**Figure 7:**
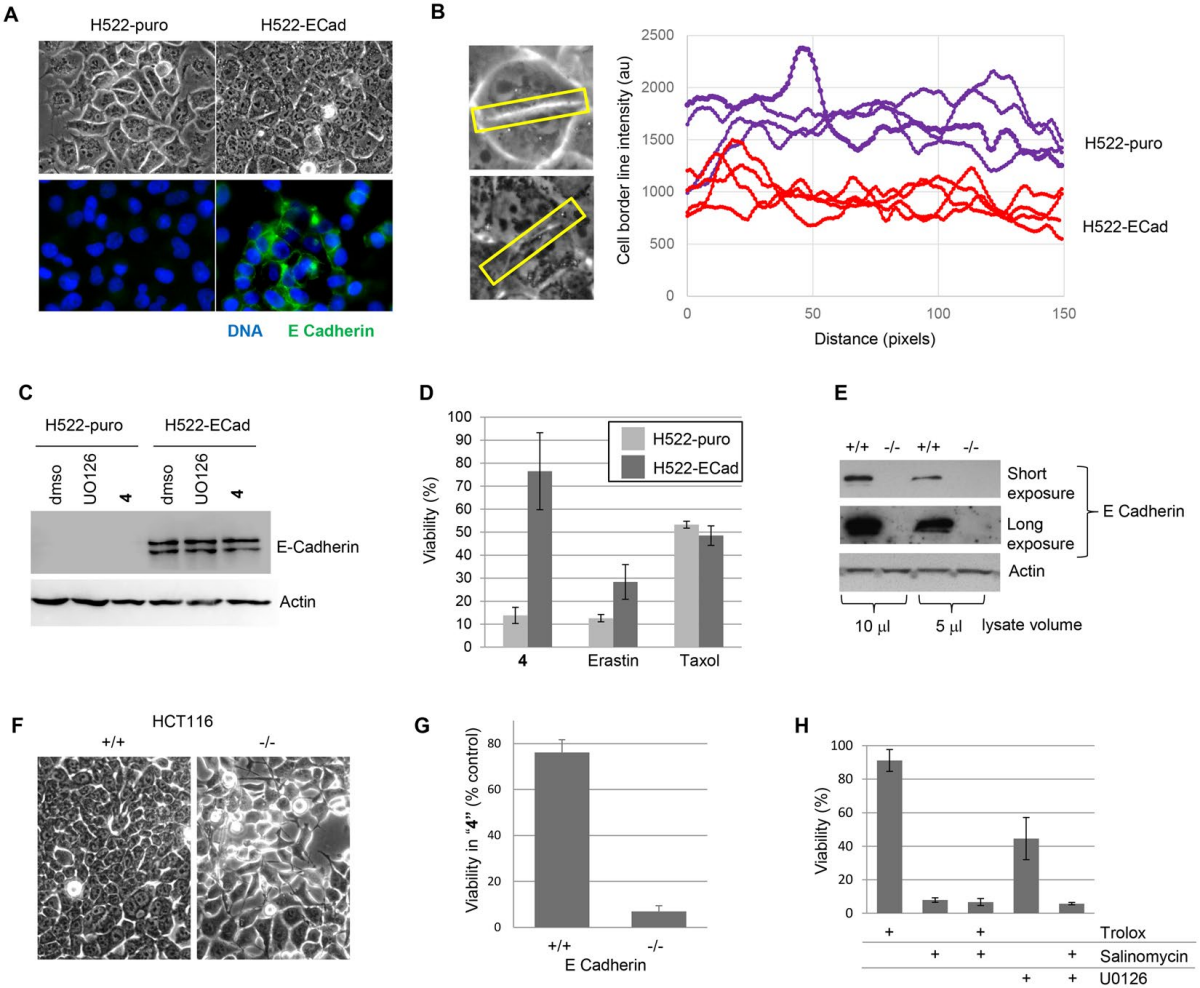
E-Cadherin modulates **4** sensitivity. (**A**) Phase contrast and immunofluorescence imaging. E-Cadherin was re-expressed in NCI-H522 as described in the methods. Phase contrast indicates closer cell contact in E-cadherin expressing cells. (**B**) Close contact is evident by lower pixel intensities in line scans at the position of cell contact (4 cell-cell contact regions are shown). (**C**) Western analysis of E-cadherin. Cells were exposed for 14 hours with the compounds indicated before analysis. (**D**) Effects of E-cadherin on viability. Viability was determined 2 days post drug addition. (**E**) Knock-out of E-cadherin using CRISPR. The *e-cadherin* gene was disrupted in HCT116 colon cancer cells using CRISPR; western blot of parental and E-cadherin −/− clone is shown (**F**) Phase contrast imaging of parental and E-cadherin knockout cells. (**G**) **4** sensitivity after E-cadherin knockout. Wild-type and E-cadherin −/− HCT116 cells were exposed to 20 μM **4** for three days. Viability was measured using methylene blue staining. (**H**) Effect of salinomycin on NCI-H522 cells. Cells were exposed to the compounds indicated and viability determined **4** days later.

**Figure 8:**
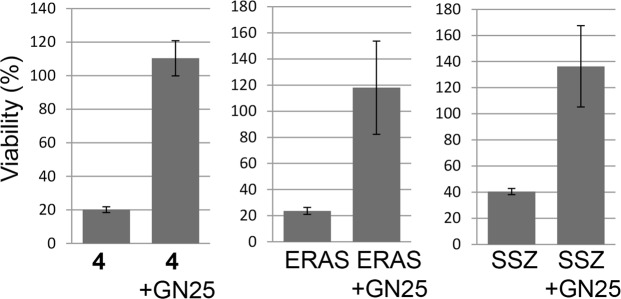
Effect of the Snail inhibitor GN25 on compound **4** toxicity. NCI-H522 cells were exposed to 10 μM GN25 for 3 days before exposing to either compound **4**, Erastin (ERAS) or sulfasalazine (SSZ). Viability was determined 2 days later using methylene blue.

Next, we re-expressed E-cadherin in the sensitive NCI-H522 cell line that lacks expression of this protein (Fig. 7A–C). E-Cadherin expressing NCI-H522 cells exhibited an epithelial growth pattern *in vitro*, unlike the parental cells, which showed less cell-cell contact (Fig. 7A,B). Exposure of reconstituted cells to either U0126 or **4** did not affect expression of exogenous E-cadherin (Fig. 7C). E-Cadherin reduced killing of NCI-H522 by **4**, as well as erastin, but had no effect on killing by taxol (Fig. 7D). Next, we used CRISPR to generate an E-cadherin knock-out version of HCT116 cells (Fig. 7E,F). Parental HCT116 cells were relatively resistant to **4**, whereas the E-cadherin-null variant cell line was killed by this compound (Fig. 7G). These results suggest that the mesenchymal state sensitizes cells to ferroptosis. Salinomycin is an antibacterial ionophore that preferentially kills mesenchymal cells possibly by modulating Wnt signaling^29,30^. Given that both salinomycin and compound **4** toxicity are reduced by expression of E-cadherin, we tested whether salinomycin might induce ferroptosis. NCI-H522 cells were sensitive to salinomycin; however, trolox did not rescue these cells (Fig. 7H). U0126 on its own reduced viability by ~50% but had no effect on salinomycin cytotoxicity (Fig. 7H). Altogether, these observations suggest that salinomycin does not kill cells by ferroptosis. In some tumor cell types, E-cadherin expression is reduced by overexpression of the transcriptional repressor Snail^31^ Exposing NCI-H522 to the Snail inhibitor GN25^32^ also blocked killing by compound **4**, however we did not detect E-cadherin re-expression (Fig. 8; our unpublished data). Therefore, it is possible that additional Snail targets modulate ferroptosis.

**Figure 9:**
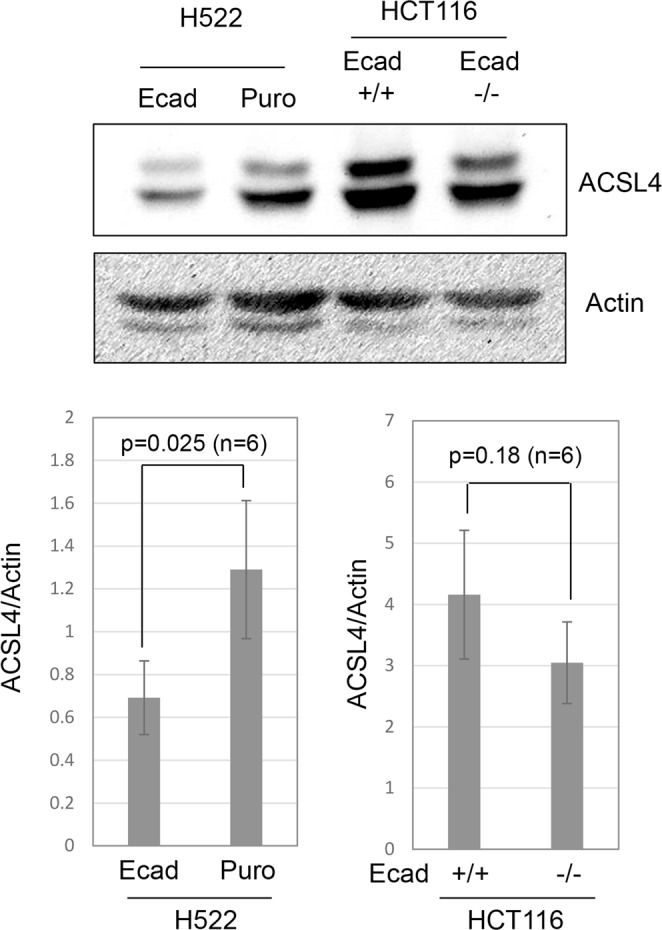
Modulation of ACSL4 levels by E-cadherin. Western blotting was used to measure ACSL4 in the indicated cell lines. Actin was used a loading control and the average ratio of ACSL4/Actin from 6 separate experiments is shown (4 independent lysates).

Next, we investigated the potential mechanism by which mesenchymal cells were sensitized to ferroptosis. First, we used western blotting to measure levels of the x_c_^−^ subunit SCL7A11. We observed no obvious change in SLC7A11 when E-cadherin was re-expressed in NCI-H522 or when it was knocked out of HCT116 (our unpublished data). We also tested the level of CBS1, an enzyme in the transulfuration pathway which might provide cysteine via modification of methionine. Modulating E-cadherin had no obvious effect on CBS1 expression (our unpublished data). Finally, we tested the level of ACSL4, a fatty acid-CoA ligase especially important in metabolism of arachidonic acid. ACSL4 sensitizes to ferroptosis by altering the lipid landscape of cellular membranes^33–36^. Re-expressing E-cadherin in NCI-H522 significantly reduced ACSL4 expression consistent with the ferroptotic resistance observed (Fig. 9). However, there was no significant difference upon knocking out E-cadherin in HCT116 (Fig. 9). These results suggest that modulating E-cadherin can alter ACSL4 expression depending on the cellular context.

### Selective killing of breast CSCs with compound 4

**Figure 10:**
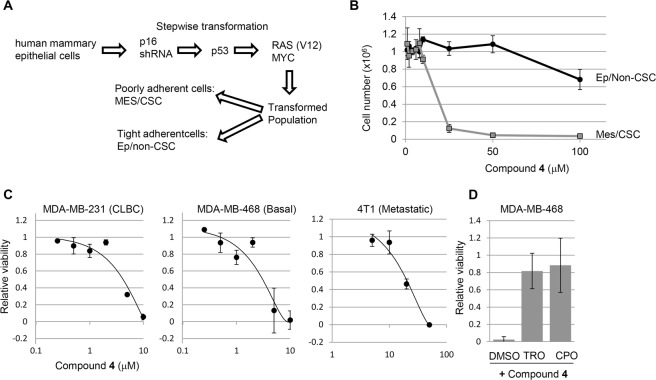
Breast CSCs and mesenchymal breast cancer are sensitive to **4**. (A&B) Cells with CSC properties are sensitized to compound **4**. (**A**) Stepwise generation of human breast cancer cells with defined genetic alterations. (**B**) Cells were exposed to compound **4** at the doses indicated 2 days post-plating. Direct cell counts were performed 2 days later. Significant differences were observed at 25 μM (p = 0.0003) 50 μM (p = 0.0003) and 100 μM (p = 0.01). Results are representative of three independent experiments. (**C**) Compound **4** dose response curves of the indicated human (231, 468) and mouse (4T1) breast cancer cell lines. (**D**) Compound **4** toxicity is blocked by trolox (TRO) and ciclopiroxolamine (CPO) in 468 cells.

An important implication of our results with E-cadherin expression is related to the CSC hypothesis. This hypothesis suggests that a subpopulation of cells within a tumor is responsible for seeding metastatic deposits and driving tumor relapse after treatment^37,38^. Some studies suggest that CSC exhibit mesenchymal properties^39,40^. Further, CSC-like cells are more difficult to kill using traditional chemotherapy^37,38^. Therefore, we tested whether **4** had differential effectiveness towards CSC in a genetically well-defined model of human breast cancer. Human mammary epithelial cells were previously neoplastically transformed by stepwise introduction of defined genetic events (activated Ras + c-Myc + p53shRNA and p16shRNA)^41^. The resulting transformed population contained epithelial and mesenchymal cells. Further, the mesenchymal but not the epithelial cells were capable of forming tumors in immunodeficient mice and expressed many markers associated with the CSC phenotype^41^. Side-by side comparison showed the mesenchymal population to be up to 20 fold more sensitive than the epithelial population to compound **4** (Fig. 10A,B). Therefore, **4** exhibits selective toxicity toward human mammary CSCs. Of the intrinsic subtypes of breast cancer, 10–15% are characterized by the expression of mesenchymal and stem cell makers^42^. These “claudin-low” tumors are sensitive to the x_c_^−^ inhibitor sulfasalazine^43^. Given that compound **4** could selectively kill mesenchymal breast cancer cells, we tested the claudin-low cell lines SUM159 and MDA MB 231, along with the basal subtype cell line MDA MB 468. Both MDA MB 231 and MDA MB 468 were highly sensitive to **4**, while SUM159 was not affected at the concentrations tested (Fig. 10C and ref.^11^). Trolox and CPO protected MDA MB 468 from **4** suggesting that cell death is due to ferroptosis (Fig. 10D). In addition, MDA MB 231 were killed by erastin consistent with the sensitivity of these mesenchymal breast cancer cells to ferroptosis (Supplementary Fig. S1). Consistent with the effects of **4** in human breast cancer, the metastatic mouse mammary cancer cell line 4T1 was also sensitive to this compound (Fig. 10C). Therefore, a subset of claudin-low and basal breast cancers may be sensitive to **4** and related compounds in a clinical setting.

## Discussion

Certain human cancers are often detected only at an advanced stage, with current therapies unable to stop the disease. For example, most (~85%) lung cancers are only detected after they have spread outside of lung and as a consequence 5 year survival rates are only ~17%. Metastatic spread and tumor relapse after therapy are critical problems with a wide range of solid tumors. Therefore, new systemic therapies are needed that can selectively destroy metastatic deposits. In our studies of open chain epothilones we have identified a class of small molecules that kill a number of human cancer cell lines. Further investigation suggests that these compounds selectively kill mesenchymal cells and cells with CSC properties. Since CSCs have been implicated in metastasis, drug resistance, and disease relapse, the class of compounds we describe may provide a novel foothold in the therapeutic approach to cancer.

The compounds we have identified appear to harness ROS created in an iron dependent manner to kill cells. Several additional features suggest that these compounds induce ferroptosis. Ferroptosis was originally defined as a mechanism of death caused by drugs selected based on preferential killing of Ras-transformed tumor cells^3^. Targeting Ras-transformation is a potentially attractive tumor-specific alteration that might allow a high degree of tumor specificity. Ras-transformation may predispose to ferroptosis by reducing antioxidant capacity^4^. Similar to other RSL molecules, compound **4**-induced killing of HT1080 harboring mutant N-Ras was reduced by inhibiting MEK1/2 with U0126. Even though NCI-H522 cells contain wild-type Ras genes, they were still sensitive to **2**, but less so when MEK1/2 was inhibited. Many human tumors acquire elevated Ras-MAPK signaling, not by mutation of Ras genes, but by mutation and/or overexpression of growth factor receptors (ex. ERBB2)^44^. Therefore, our results suggest that RSL molecules, like erastin and **4**, may be effective against a wider range of tumors than originally thought, with the crucial feature being elevation of Ras-MAPK signaling which can occur in a number of ways. As noted earlier, since U0126 also has antioxidant activity, the exact role of RAS-MAPK signaling in **4** cytotoxicity remains to be directly determined. Several additional RSL compounds that induce ferroptosis have been recently identified^2,4,5^. Although structurally diverse, many of these compounds can be classified as Type I compounds that block cystine uptake leading to glutathione depletion or Type II compounds that directly inhibit GPX4^5^. Compound **4** depleted glutathione, reduced both glutamate secretion and cystine-FITC uptake, and killed cells in a manner that was blocked by either βME or N-acetylcysteine. All of these features strongly suggest that our compounds are Type I inhibitors that directly block cystine uptake, possibly by direct binding to the x_c_^−^ transporter.

In comparing sensitivities of various tumor cell lines in cell culture studies, we found a number of mesenchymal cells among the sensitive group. Also, among 60 cell lines tested in an NCI screen, only those with low E-cadherin (a characteristic of the mesenchymal state) were sensitive to **1**. Further, re-expression of E-cadherin in NCI-H522 reduced killing by compound **4** while knocking E-cadherin out of HCT116 cells sensitized them. The mechanism by which E-cadherin suppresses ferroptosis is currently unknown. One clue that we uncovered is that ACSL4 expression was lower when E-cadherin was re-expressed in NCI-H522 cells. Therefore, depending on the cellular context, modulating E-cadherin may alter ferroptosis by altering the lipid composition of cellular membranes.

For many years, loss of E-cadherin has been known to occur during progression to more advanced cancer stages^45^. Therefore, the compounds we have identified may provide a novel approach to treat aggressive tumors. The observation that **4** and other ferroptosis-inducers target aspects of the mesenchymal state suggest that these compounds may represent a new approach to kill CSCs. Consistent with this idea, transformed human mammary epithelial cells that acquired mesenchymal CSC features were 20 fold more sensitive than epithelial cells transformed with the same genetic alterations. In addition to our genetically-defined breast cancer model, two additional breast cancer cell lines, MDA MB 231 and MDA MB 468 were sensitive to **4**. MDA MB 231 is a mesenchymal cell line representative of the “claudin-low” subtype providing additional evidence that **4** targets mesenchymal cells. However, MDA MB 468 are a basal subtype that expresses E-cadherin. Of note, MDA MB 468 express stem cell markers CD133 and CD44^46^. Therefore, sensitivity is likely more complicated than a simple EMT switch. Instead, a critical combination of stem and mesenchymal markers may render cells sensitive to 4-induced ferroptosis.

An additional implication of our findings is that ferroptosis may be particularly helpful in the treatment of sarcomas derived from mesenchymal tissues. Consistent with this idea, we found that HT1080 fibrosarcoma, Rh30 and Rh41 rhabdomyosarcoma, and TC71 Wilm’s tumor cell lines were sensitive to **4**^11^. Altogether, our observations point to a new therapeutic approach to cancer. Limitations of our study include the fact that the mechanism by which E-cadherin and EMT sensitize to the compounds is not known. In addition, several tumor cell lines tested are relatively resistant to the compounds. Therefore, patients with tumors that do not contain a mesenchymal or stem cell subpopulation may not be helped by these compounds. Before these compounds can be used in the clinic, detailed efficacy and toxicity studies in animal models must be carried out, followed by toxicity and efficacy trials in humans. In addition, SAR analysis should be continued to enhance specific toxicity and enhance pharmacokinetic properties.

## Conclusion

During the course of our studies to develop structurally simplified epothilone analogues, we discovered a new class of small molecules that kill selected cancer cell lines by a novel mechanism of action. The cytotoxic activity is cell cycle-independent and does not involve apoptosis. The compounds had no effect on tubulin or mitochondrial function. They harness reactive oxygen species to kill cancer cells by ferroptosis, a recently reported iron-dependent mechanism of action. Studies are in progress to determine the specific molecular targets of these promising “drug-like” small molecule anticancer agents.

## Materials and Methods

### Synthesis

The test compounds were synthesized as described by us recently^11^.

### Cell Lines and Culture Conditions

Cell lines were cultured in a humidified 37 °C atmosphere containing 10% CO_2_ in Dulbecco’s Modified Eagle’s Medium (Mediatech, Inc.) supplemented with 10% fetal bovine serum (Atlanta Biologicals) and 1000 U/ml of both Penicillin and Streptomycin (Mediatech, Inc). Cell lines were obtained from the following sources: NCI-H522 and HOP62 (National Cancer Institute), WI38 (ATCC), MDAH041 (Michael Tainsky, Wayne State University), MDA MB 231 and MDA MB 468 (Song-Tao Liu, University of Toledo), 4T1 (Kam Yeung, University of Toledo), RPE (Prasad Jallepalli, Memorial Sloan Kettering), HCT116 (Bert Vogelstein, Johns Hopkins), SUM159 (Rafael Garcia-Mata, University of Toledo) HT1080 (George Stark, Cleveland Clinic). Mes/Ep cell populations were generated as previously described^41^. To re-express E-Cadherin in NCI-H522, we used the retroviral vector “pWZL Blast mouse E-cadherin” (Addgene #18804). Retroviral DNA was transiently transfected into the Phenix amphotropic packaging cell line to create viral particles. Cell culture supernatant was filtered and used to infect NCI-H522. Twenty blasticidin-resistant clones were pooled to reconstitute E-Cadherin expression. To knockout E-Cadherin, the following oligonucleotides (5′-CACC GCC CTT GGA GCC GCA GCC TCT-3′-TOP; 5′-AAA CAG AGG CTG CGG CTC CAA GGG C-3′-BOTTOM) were ligated into px459, which also expresses Cas9. (Addgene #62988) Ligations were carried out as described^47,48^ and products were confirmed by sequencing. p53-knockout HCT116 cells were transfected using GenePorter (GenLantis), clones selected using 1 μg/ml of puromycin and screened by western blotting with an antibody to E-Cadherin. To determine viability 50,000 cells were plated in each well of a 24-well plate. Compounds were added 1 day post plating and cells were stained after 3 days with a saturated solution of methylene blue in 50% ethanol. Plates were rinsed, retained dye was dissolved in 0.1N HCl and absorbance measured at 668 nm. Results are representative of at least two independent experiments. Some experiments were repeated using MTS dye (CellTitre96, Promega) with similar results.

### Microscopy

For immunofluorescence microscopy, cells were fixed with 2% formaldehyde in phosphate buffered saline (PBS) for 10 minutes, followed by permeabilization [150 mM NaCl, 10 mM Tris (pH 7.7), 0.1% Triton X-100, and 0.1% BSA] for 9 minutes^49^. Fixed cells were blocked with PBS containing 0.1% BSA for 1 h at room temperature. Cells were then stained with antibodies to E-Cadherin (Cell Signaling Technology). Antibodies were visualized by incubating samples with Alexafluor-conjugated secondary antibodies (Invitrogen). DNA was visualized by staining with Hoechst 33342. Images were captured using Photometrics Coolsnap HQ2 camera connected to an Olympus IX81 inverted microscope.

### Western blotting

Cell pellets were lysed in RIPA buffer containing 10 mM Tris (pH 7.4), 150 mM NaCl, 1% NP-40, 1% DOC, 0.1% SDS (supplemented with 1 µg/ml aprotinin, 2 µg/ml leupeptin, 1 µg/ml pepstatin A, 1 mM DTT, and 0.1 M PMSF) for 20 minutes on ice and centrifuged at high speed for 20 minutes at 4 °C. Extracts containing equal quantities of proteins, determined by using the BCA Protein Assay Kit (Pierce) were separated by SDS-polyacrylamide gel electrophoresis (12.5% acrylamide) and transferred to polyvinylidene difluoride membranes (Millipore). Membranes were incubated in blocking buffer [5% (w/v) non-fat dry milk dissolved in PBST (1X PBS containing 0.05% [v/v] Tween 20)] for 1 hour at room temperature. Membranes were then incubated with antibodies to phosphorylated ERK (Cell Signaling), E-Cadherin (Cell Signaling), ACSL4 (Thermo), or Actin (Abcam) diluted 1:1000 in blocking buffer for 1–2 hours at room temperature. Secondary antibodies conjugated to horse-radish peroxidase were obtained from Biorad and used at a dilution of 1:10,000. Bound antibodies were detected using enhanced chemiluminescence (Biorad).

### ROS Measurement

To measure ROS, 10,000 NCI-H522 cells were plated into each well of a black, sterile 96-well plate in a volume of 200 µl. Compounds were added for 8 hours before ROS were detected. To detect ROS, H2DCFDA was dissolved at 500 µM in dimethyl sulfoxide (DMSO). This H2DCFDA was diluted 1:10 in DMEM, and 50 µl added to each well of the 96-well plate without removing the growth medium. Cells were then incubated for 30 minutes at 37 °C. Cells were washed twice with PBS and then 100 µl of PBS added to each well before fluorescence was measured. Plates were analyzed in a multi-well plate reader using excitation at 485 nm and emission at 520 nm. Alternatively, after two PBS washes, dye was extracted using 20% (w/v) sodium dodecyl sulfate following by fluorescence measurement in the plate. Similar results were obtained with or without dye extraction. For analysis of lipid ROS, NCI-H522 cells were treated with DMSO or compound 4 (10 μM). Bodipy 581/591 C11 (0.2 μM) was added at the time of treatment. Ten hours later cells were collected by trypsinization, washed once with PBS and resuspended in PBS containing 2% FBS. Cells were analyzed using a BD LSR Fortessa FACScanner and FlowJo software. Thirty thousand cells were analyzed for each sample.

### Glutathione Measurement

Glutathione was measured using monochlorobimane (MCB). First, a stock of MCB was prepared by dissolving 1.8 mg of MCB powder in 1.98 ml DMSO (4 mM). This stock was stored in 200 µl aliquots at −20 °C. To stain cells, 200 µl of 4 mM MCB was dissolved in 25 ml of PBS (final concentration 32 µM) and 200 µl of this dilution added to cells plated in a black 96-well plate. In a typical experiment, 10,000 cells were plated per well and treated with test compounds for 8 or 16 hours before glutathione levels measured. Once the dye was added, the cells were incubated for 30 minutes at 37 °C. Fluorescence was measured using a plate reader with excitation set at 400 nm and emission set at 500 nm. Glutathione was also measured using the Grx1-roGFP2 biosensor as follows: 30,000 HT1080 cells were plated in a 24 well plate and transfected with Grx-roGFP2 24 hours later (Addgene # 64975). Transfections were carried out using lipofectamine 3000 according to the manufacturers instructions (Thermo). Thirty six hours after transfection, 20 μM compound **4** was added and confocal imaging performed 8 hours later. Imaging involved scanning with a 405 nm laser and collecting a single z-plane using a 510nm–570nm window. The same cell was immediately scanned using 488 nm excitation and signal detected using the same 510nm–570nm emission window. All images were collected using an environmental chamber to keep cells at 37 °C. pH was maintained by adding 25 mM HEPES buffer (pH 7.6)^50^.

### Measurement of Glutamate Secretion

Glutamate released into the culture medium was measured using the Amplex Red Glutamic Acid Kit (Invitrogen). Specifically, an Amplex Red reaction mixture was prepared containing glutamate oxidase, L-alanine, L-glutamate-pyruvate transaminase, horse radish peroxidase and Amplex red reagent. In this mixture, glutamate oxidase converts L-glutamate from the culture medium into α-ketoglutarate, NH_3_, and H_2_O_2_. The α-ketoglutarate is converted back to L-glutamate using L-alanine and L-glutamate-pyruvate transaminase to allow multiple cycles of L-glutamate oxidation and amplification of H_2_O_2_ production. Horse radish peroxidase uses H_2_O_2_ to convert Amplex red to the fluorescent product resorufin. The amount of resorufin depends on the initial input of L-glutamate from the sample measured. To perform the assay, 25,000 NCI-H522 cells were plated into each well of a 24-well plate in a volume of 500 µl. One day later, the cells were treated with test compounds for 3 hours. Next, the medium was removed, and wells washed 3 times with prewarmed DMEM. Two hundred µl of DMEM containing test compounds was added to each well, and 50 µl samples were removed at increasing time intervals. Twenty five µl of the sample was placed in a well of a 96-well plate along with 25 µl of Amplex Red reaction mixture described above and prepared as per the manufacturers instructions. Reaction proceeded for 30 minutes at 37 °C after which fluorescence emission was measured at 590 nm using excitation between 530 and 560 nm.

### Measurement of Cystine-FITC uptake

Uptake of cystine-FITC was measured as follows: 25,000 MDA MB 231 cells were plated in 24 well plates and treated 24 hours later with compound **4** at 20 μM. To improve viability with this concentration of compound, liproxstatin was also added at 2.5 μM. Sixteen hours later, cells were washed with Choline chloride buffer (137 mM choline chloride [pH 7.4], 3 mM KCl, 1 mM CaCl_2_, 2 mM D-Glucose, 0.7 mM K_2_HPO_4_ and 10 mM HEPES) and starved in the same buffer for 30 minutes at 37 °C. Cystine-FITC (EMD Millipore) was added at a final concentration of 5 μM for 1 hours. Cells were washed twice with Choline buffer and FITC fluorescence detected using a EVOS imager. Drugs were present during the final washes and in the imaging buffer. Digital images were collected and quantified using ImageJ.

## Supplementary information

Supplementary Figures

dataset 1

## Data Availability

All datasets, cell lines and reagents are freely available to the research community upon request.
