# Supplementary material for: Small-Molecule Ferroptotic Agents with Potential to Selectively Target Cancer Stem Cells
Source: Sci Rep. 2019 Apr 11;9:5926. doi: 10.1038/s41598-019-42251-5 (PMC6459861; doi:10.1038/s41598-019-42251-5)
Supplement: Supplementary file 1 — Supplementary Figures [file 41598_2019_42251_MOESM1_ESM.pdf]

Small-Molecule Ferroptotic Agents with Potential to Selectively Target Cancer Stem Cells

William R. Taylor[†]*, Sara R. Fedorka[§], Ibtissam Gad[†], Ronit Shah[†], Hanan D. Alqahtani[†], Radhika Koranne[†], Nishanth Kuganesan[†], Samkeliso Dlamini[§], Tim Rogers[†], Ayad Al-Hamashi[§], Veronika Kholodovych[†], Yusuf Barudi[†], Damian Junk[£], Maisha S. Rashid[†], Mark W. Jackson[£], and L. M. Viranga Tillekeratne[§]*

[§]Department of Medicinal and Biological Chemistry, College of Pharmacy and Pharmaceutical Sciences, The University of Toledo, 2801, W. Bancroft Street, Toledo, Ohio 43606, United States
[†] Department of Biological Sciences, College of Natural Sciences and Mathematics, The University of Toledo, 2801, W. Bancroft Street, Toledo, Ohio 43606, United States
[£]Department of Pathology, Case Western Reserve University School of Medicine, Case Comprehensive Cancer Center, 2103 Cornell Road, WRB 3-134, Cleveland, OH 44106
*These authors contributing equally to this work and are co-corresponding authors.

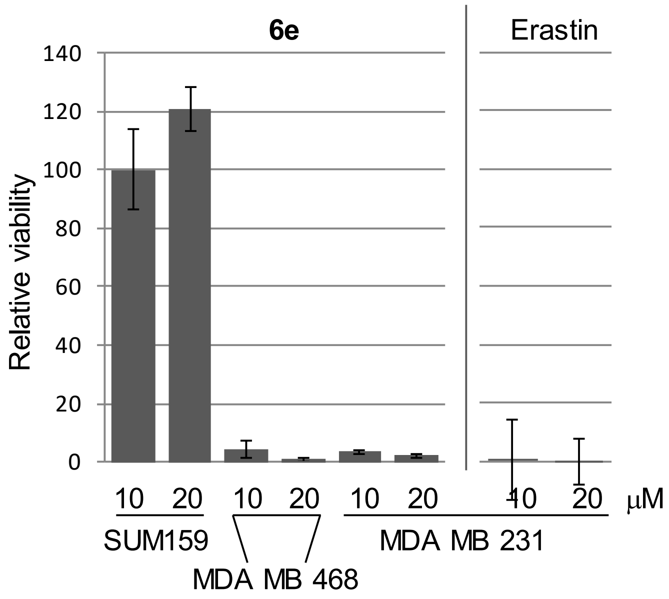

Figure S1. Effect of 4 and Erastin on human breast cancer. Cell lines were exposed to drugs for 2 days and then viability determined. To determine viability, cells were stained with methylene blue.

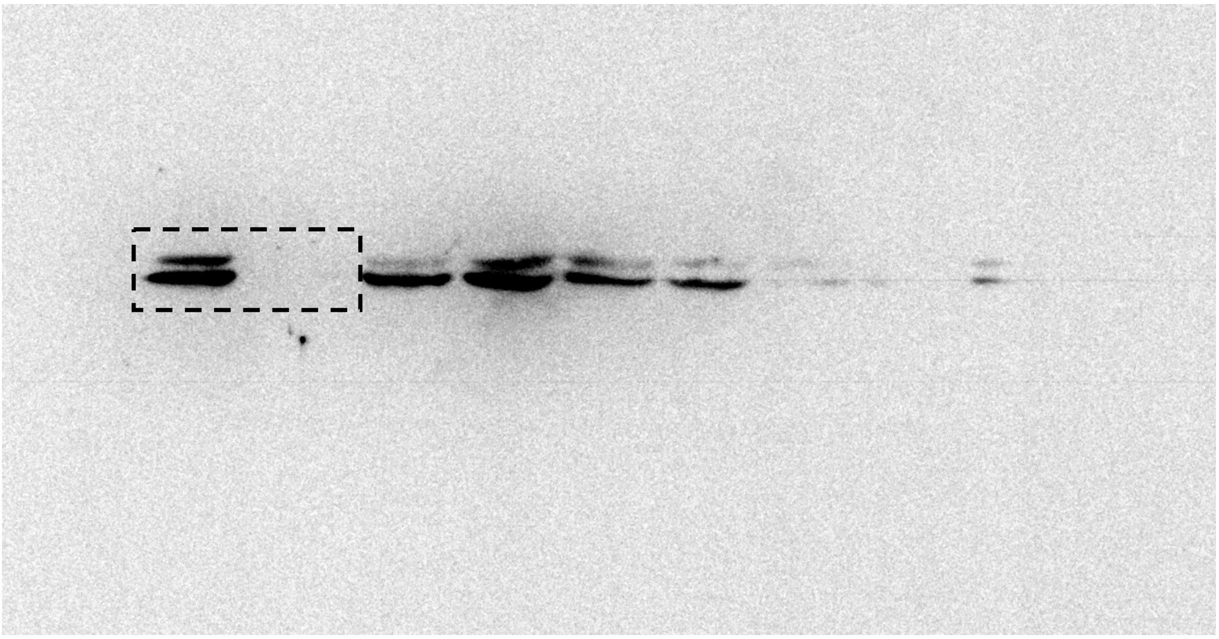

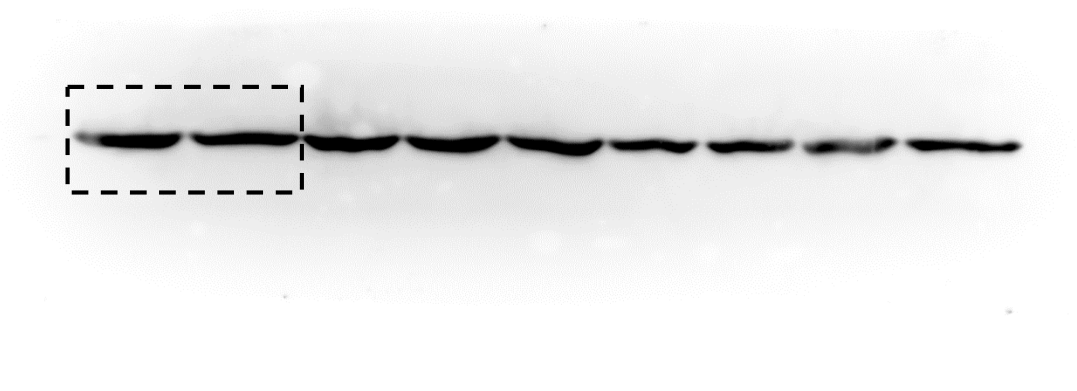

Figure S2. Full blots used in figure 2D indicating cropping.

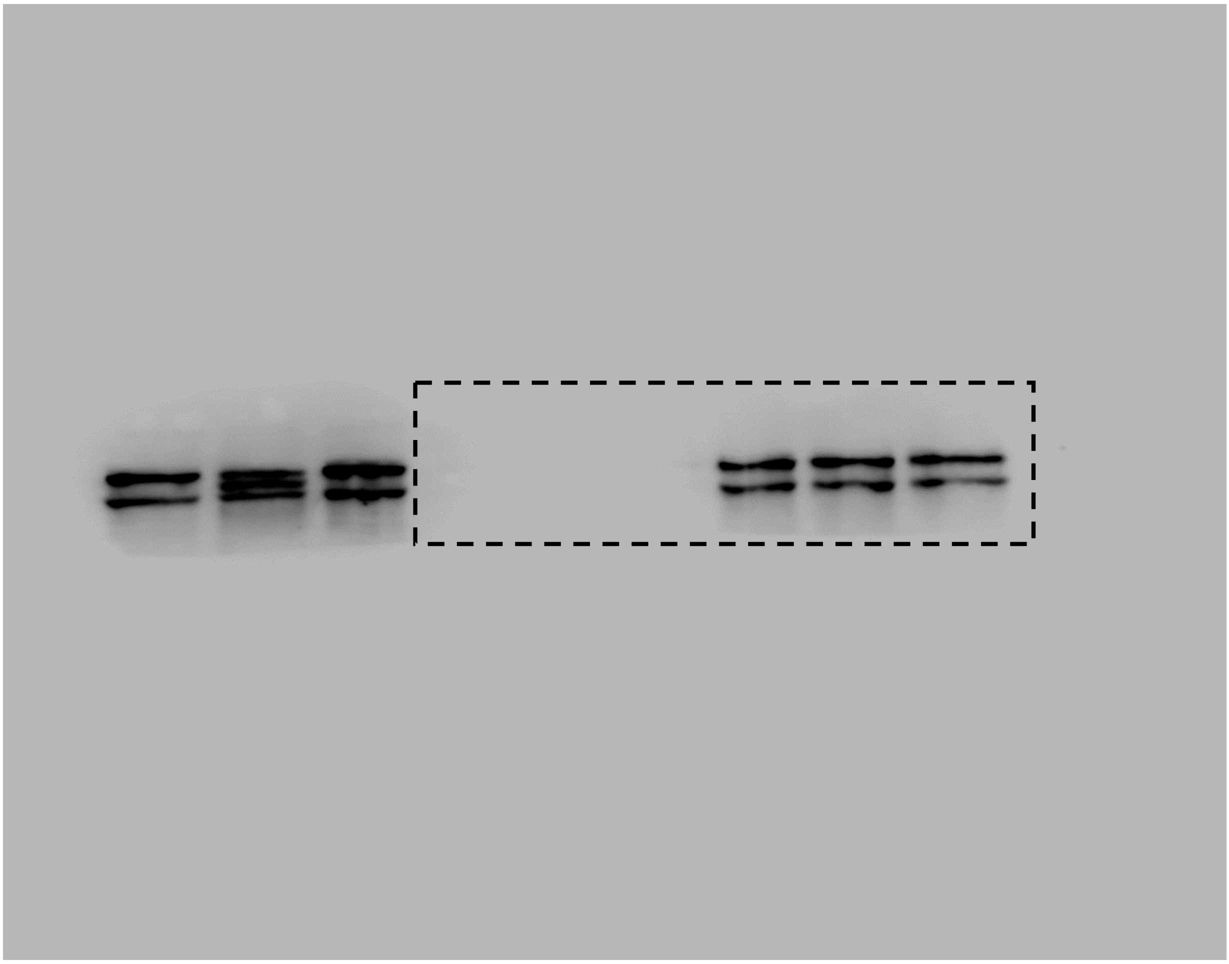

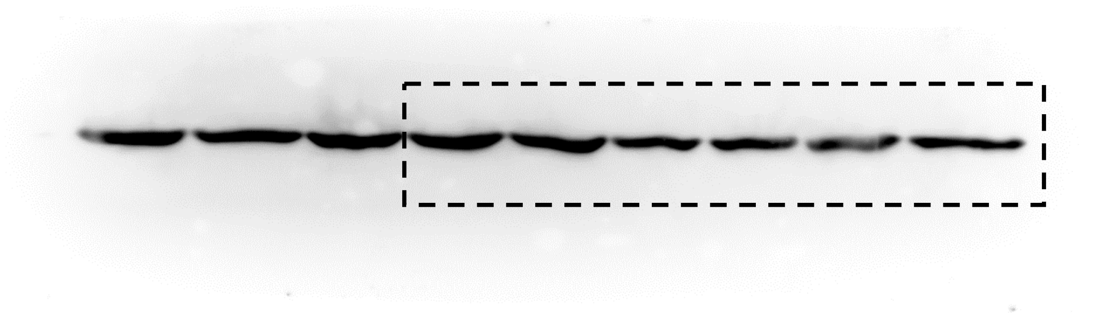

Figure S3. Full blots used in figure 7C indicating cropping.

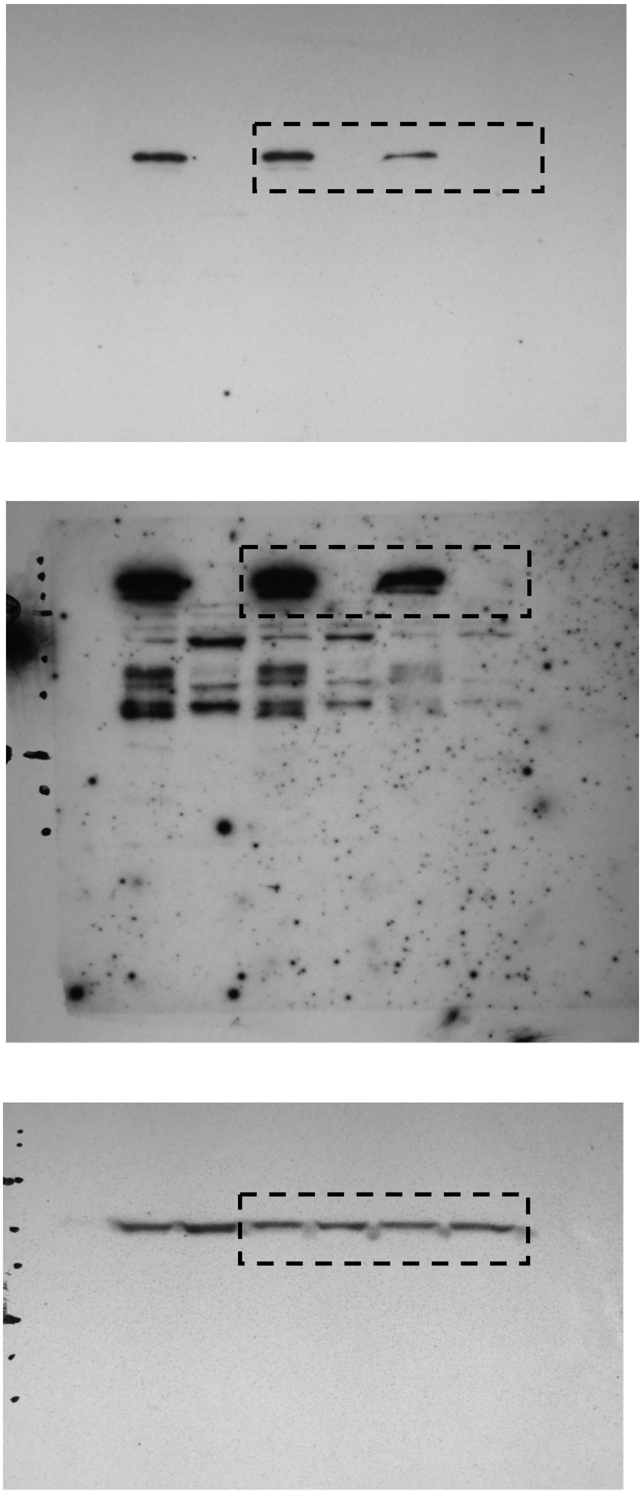

Figure S4. Full blots used in figure 7E indicating cropping.

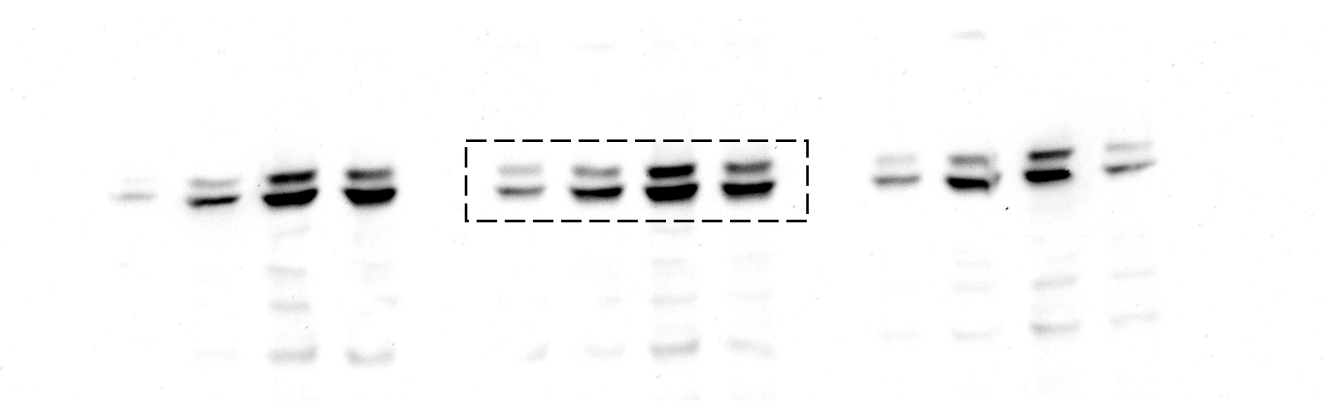

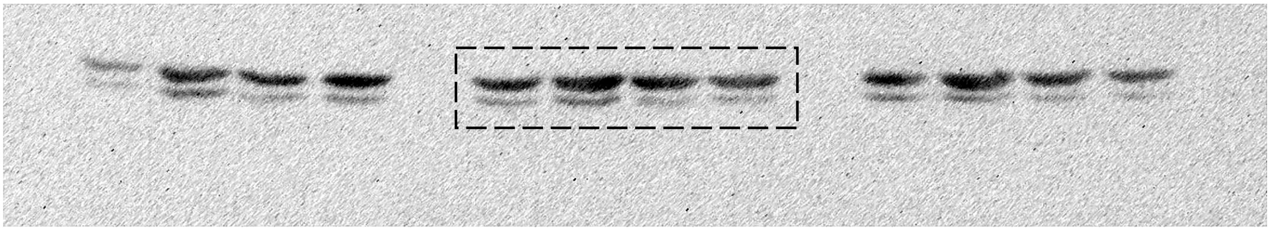

Figure S5. Full blots used in figure 9 indicating cropping.
